# Vitellogenin Underwent Subfunctionalization to Acquire Caste and Behavioral Specific Expression in the Harvester Ant *Pogonomyrmex barbatus*

**DOI:** 10.1371/journal.pgen.1003730

**Published:** 2013-08-15

**Authors:** Miguel Corona, Romain Libbrecht, Yannick Wurm, Oksana Riba-Grognuz, Romain A. Studer, Laurent Keller

**Affiliations:** 1 Department of Ecology and Evolution, University of Lausanne, Lausanne, Switzerland; 2 Bee Research Laboratory USDA-ARS, Beltsville, Maryland, United States of America; 3 Laboratory of Insect Social Evolution, Rockefeller University, New York City, United States of America; 4 School of Biological and Chemical Sciences, Queen Mary University of London, London, United Kingdom; 5 Institute of Structural and Molecular Biology, Division of Biosciences, University College London, London, United Kingdom; University of Michigan, United States of America

## Abstract

The reproductive ground plan hypothesis (RGPH) proposes that the physiological pathways regulating reproduction were co-opted to regulate worker division of labor. Support for this hypothesis in honeybees is provided by studies demonstrating that the reproductive potential of workers, assessed by the levels of vitellogenin (Vg), is linked to task performance. Interestingly, contrary to honeybees that have a single Vg ortholog and potentially fertile nurses, the genome of the harvester ant *Pogonomyrmex barbatus* harbors two Vg genes (*Pb_Vg1* and *Pb_Vg2*) and nurses produce infertile trophic eggs. *P. barbatus*, thus, provides a unique model to investigate whether Vg duplication in ants was followed by subfunctionalization to acquire reproductive and non-reproductive functions and whether Vg reproductive function was co-opted to regulate behavior in sterile workers. To investigate these questions, we compared the expression patterns of *P. barbatus* Vg genes and analyzed the phylogenetic relationships and molecular evolution of Vg genes in ants. qRT-PCRs revealed that *Pb_Vg1* is more highly expressed in queens compared to workers and in nurses compared to foragers. By contrast, the level of expression of *Pb_Vg2* was higher in foragers than in nurses and queens. Phylogenetic analyses show that a first duplication of the ancestral Vg gene occurred after the divergence between the poneroid and formicoid clades and subsequent duplications occurred in the lineages leading to *Solenopsis invicta*, *Linepithema humile* and *Acromyrmex echinatior*. The initial duplication resulted in two Vg gene subfamilies preferentially expressed in queens and nurses (subfamily A) or in foraging workers (subfamily B). Finally, molecular evolution analyses show that the subfamily A experienced positive selection, while the subfamily B showed overall relaxation of purifying selection. Our results suggest that in *P. barbatus* the Vg gene underwent subfunctionalization after duplication to acquire caste- and behavior- specific expression associated with reproductive and non-reproductive functions, supporting the validity of the RGPH in ants.

## Author Summary

One of the main features of social insects is the division of labor, whereby queens monopolize reproduction while sterile workers perform all of the tasks related to colony maintenance. The workers usually do so in an age-dependent sequence: young workers tend to nurse the brood inside the nest and older workers are more likely to forage for food. Previous studies revealed that vitellogenin, a yolk protein typically involved in the regulation of reproduction in solitary insects, has been co-opted to regulate division of labor in the honeybee. In this study, we investigate such a role of vitellogenin in another group of social insects: the ants. We first use phylogenetic analyses to reveal the existence of multiple vitellogenin genes in most of the sequenced ant genomes. Then we compare the expression of the two vitellogenin genes (*Pb_Vg1* and *Pb_Vg2*) among queens, nurses and foragers in the seed-harvester ant *Pogonomyrmex barbatus*. Our results suggest that, after the initial duplication in ants, the vitellogenin genes acquired caste and behavioral specific expression associated with reproductive and non-reproductive nutritionally related functions. This study also shows that ants and bees, despite having evolved sociality independently, have conserved similar mechanisms to regulate division of labor.

## Introduction

Division of labor is the cornerstone of insect societies and implies the coexistence of individuals that differ in morphology, reproduction and behavior [1], [2]. There are usually two levels of division of labor among individuals in social insect colonies. The first relates to a reproductive division of labor, whereby reproduction is monopolized by one or several queens while sterile workers perform all the tasks related to colony maintenance. The second relates to a division of labor among the worker force, whereby workers perform different tasks in an age-dependent sequence: young workers usually perform tasks inside the colony (e.g. brood care) while old workers forage outside the nest [3], [4]. The colony organization of advanced eusocial insects evolved independently in ants, bees, and wasps [5], [6]. While the ecological constraints favoring social evolution are well studied [7], it remains largely unknown whether the genetic mechanisms regulating behavior are conserved among species [8]–[12].

The ovarian ground plan hypothesis (OGPH) is a theoretical framework that seeks to explain the evolution of reproductive division of labor in social insects [13], [14]. The OGPH proposes that the physiological pathways regulating the reproductive and behavioral cycles of solitary ancestors have been co-opted and selected to evolve into the queen and worker castes of existing eusocial insects. This hypothesis is based on the observations that the ovarian cycle of alternate development and depletion phases of solitary wasps is associated with reproductive and non-reproductive behavioral traits that resemble the queen and worker castes of eusocial insects: females with developed ovaries lay eggs while females with undeveloped ovaries forage for food and defend the nest [13]. The same was likely true in the solitary ancestors of ants and bees. The reproductive ground plan hypothesis (RGPH) extends this concept to explain the evolution of worker division of labor in honeybees [15]–[17]. Indeed, honeybee worker subcastes have two distinctive phases of ovarian activity, with nurses having large ovaries and high titers of the yolk protein vitellogenin (Vg), and foragers small ovaries and low titers of Vg [15], [18], [19]. The RGPH suggests that the mechanisms controlling ovarian activity influence the behavioral development and the mechanisms of food collection in worker honeybees. Support for this hypothesis is provided from studies demonstrating that workers with larger ovaries and higher titers of Vg are more likely to forage at younger ages and show a pollen foraging bias compared to workers with smaller ovaries and lower titers of Vg, which are more likely to forage at older ages and show a nectar foraging bias [15], [16]. These variations in reproductive traits have a genetically inherited component as strains with different ovarian activity and foraging bias have been selected from wild type populations [15], [20].

Although it has been established that the pleiotropic mechanisms connecting reproduction and division of labor have a genetic component, three lines of evidence suggest that the two processes are linked by an additional nutritional factor. First, in honeybees, reproductive queens and potentially reproductive nurses (with large and medium-sized ovaries, respectively) [18], [21], [22] consume diets with higher protein content [23] compared to sterile foragers with smaller and presumably non-functional ovaries [18]. Second, pollen consumption in nurses [23] is associated with higher Vg protein levels [19], compared to foragers that only consume honey [23]. Finally, there is a causal relationship between nutrition, Vg levels and behavior as pollen consumption is required to induce Vg expression [24], [25] and experimental reduction of Vg expression results in precocious foraging [26], [27].

To determine whether the co-option of reproductive pathways plays a major role in social evolution would require to investigate the link between reproductive physiology and behavior in other social insects, preferentially in those, such as ants, that evolved sociality independently from bees [5], [6], [28]. Ants have two additional characteristics that make them a particularly interesting model to study the predictions of the RPGH. First, in contrast to the honeybee genome that contains a single Vg gene, ant genomes can harbor multiple Vg genes. Indeed, the genome of the fire ant *Solenopsis invicta* harbors four Vg genes, two of them preferentially expressed in queens (*Si_Vg2* and *Si_Vg3*) and the two others in foraging workers (*Si_Vg1* and *Si_Vg4*) [29]. Vg duplication and subsequent subfunctionalization to acquire caste-specific expression provides a unique opportunity to test whether the genes associated with reproduction were co-opted to regulate worker behavior. Second, also in contrast with bees and wasps, where workers are facultatively sterile, workers are fully sterile in a significant number of ant species, including *P. barbatus* and *S. invicta* [30]–[32], which allows one to test the hypothesis that Vg reproductive function was co-opted to regulate behavior in species with fully sterile workers.

The first aim of this study was to determine the number of Vg genes in *P. barbatus* and other ants and investigate their phylogenetic relationships. This analysis is expected to determine whether the occurrence of multiple Vg genes is a phenomenon specific to *S. invicta* [29] or shared with other ant species as well as provide information on the origin and evolution of Vg genes in ants. Our second objective was to test whether Vg genes in *P. barbatus* display caste-specific expression profiles similar to that observed in *S. invicta*, which will address the question whether Vg gene duplication and subfunctionalization to acquire caste-specific functions is a common feature in ant species. Finally, the third objective of this study was to investigate whether the expression of Vg genes *in P. barbatus* is associated with task performance as predicted by the RGPH. We carried out these objectives by annotating Vg genes, building a phylogenetic tree, measuring mRNA levels of *P. barbatus* Vg genes in queens, nurses and foragers and performing molecular evolution analyses.

## Results

**Figure 1:**
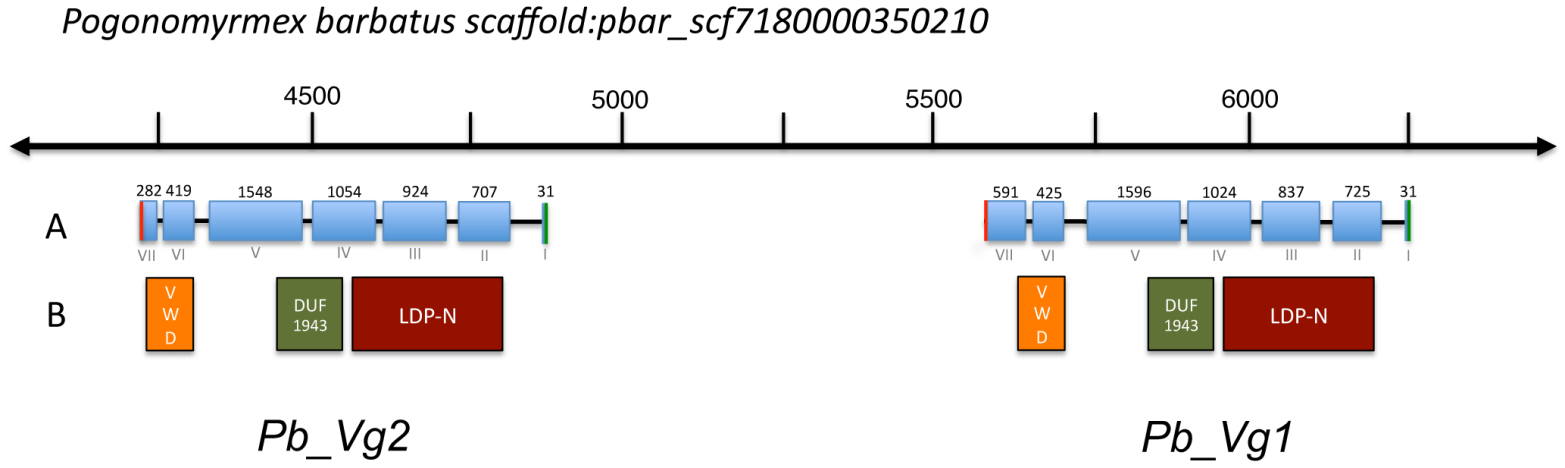
Genomic map of vitellogenin genes in *Pogonomyrmex barbatus*. A. Exon-intron organization of *Pb_Vg1* and *Pb_Vg2* genes. Blue boxes represent exons and joining lines introns. Green and red lines indicate start and stop codons respectively. Exon number (I–VII) and size (31–1596 bp) are indicated at the bottom and top of the figure. Exon VII in *Pb_Vg2* (282 bp) is truncated with respect to *Pb_Vg1* (591 bp). B. Localization of predicted protein structural domains: LPD-N (vitellogenin), DUF1943 and VDW.

We identified two adjacent copies of Vg genes (*Pb_Vg1* and *Pb_Vg2*) in the genome of *Pogonomyrmex barbatus* [33] with predicted lengths of 1742 and 1654 amino acids, respectively (Table 1). The two genes are separated by a putative mariner-like transposon, a DNA transposable element that has been involved in duplication events [34]. The two Vg genes have an identical number of exons (Figure 1A) and share the three structural domains typical of vitellogenins: the lipoprotein N-terminal domain (LPD-N), the domain of unknown function 143 (DUF1943) and the von Willebrand factor type D domain (VWD) [35], [36] (Figure 1B). However, the coding sequence of *Pb_Vg2* is truncated compared to *Pb_Vg1* because of an earlier stop codon in the last exon of *Pb_Vg2*.

**Table 1:** Predicted protein length of Vg genes in ants.

Species	Gene	Length	Subfamily	Expression Bias	GI number
*Atta cephalotes*	*Ac_Vg1*	1747	A	?	ACEP11141-PA
*Atta cephalotes*	*Ac_Vg2*	?	B	?	ACEP22565-PA
*Acromyrmex echinatior*	*Ae_Vg1*	1738	A	?	AECH16709-PA
*Acromyrmex echinatior*	*Ae_Vg2*	1655	B	?	AECH16711-PA
*Acromyrmex echinatior*	*Ae_Vg3*	1650	B	?	AECH16710-PA
*Camponotus floridanus*	*Cf_Vg*	1845	A	?	CFLO22478-PA
*Harpegnathos saltator*	*Hs_Vg*	1754	A	?	HSAL13088-PA
*Linepithema humile*	*Lh_Vg1*	1738	A	?	LH17501-PA
*Linepithema humile*	*Lh_Vg2*	1758	A	?	LH17497-PA
*Linepithema humile*	*Lh_Vg3*	1749	A	?	None
*Linepithema humile*	*Lh_Vg4*	1792	A	?	LH17495-PA
*Linepithema humile*	*Lh_Vg5*	1673	B	?	LH25675-PA
*Pogonomyrmex barbatus*	*Pb_Vg1*	1742	A	Q>N>F	PB16966-PA
*Pogonomyrmex barbatus*	*Pb_Vg2*	1654	B	F>Q = N	PB16966-PA
*Solenopsis invicta*	*Si_Vg1*	1641	B	F>Q	SINV15084-PA
*Solenopsis invicta*	*Si_Vg2*	1808	A	Q>F	SINV14922-PA
*Solenopsis invicta*	*Si_Vg3*	1761	A	Q>F	SINV14925-PA
*Solenopsis invicta*	*Si_Vg4*	1650	B	F>Q	None

Vg paralogs are divided in two subfamilies with different phylogenetic origin and protein length. Subfamilies A and B, range from 1641 to 1673 and from 1738 to 1845 amino acids, respectively. Known expression bias and gene identification numbers of the automatic gene predictions from which our gene models were derived are specified. *Ac_Vg2* genomic sequence is uncompleted.

To determine whether the presence of multiple Vg genes is a general feature of ants, we searched for Vg genes in the five additional recently published ant genomes. These are divided up into four different subfamilies: Myrmicinae (*Atta cephalotes* and *Acromyrmex echinatior*) [37], [38], Formicinae (*Camponotus floridanus*) [39] Dolichoderinae (*Linepithema humile*) [40] and Ponerinae (*Harpegnathos saltator*) [39]. We found that numbers of Vg genes vary between one and five per species (Table 1), and that when a genome contains multiple Vg genes, they are always adjacent. To determine the evolutionary history of these genes, we subsequently constructed a phylogenetic tree using known hymenopteran Vg sequences.

**Figure 2:**
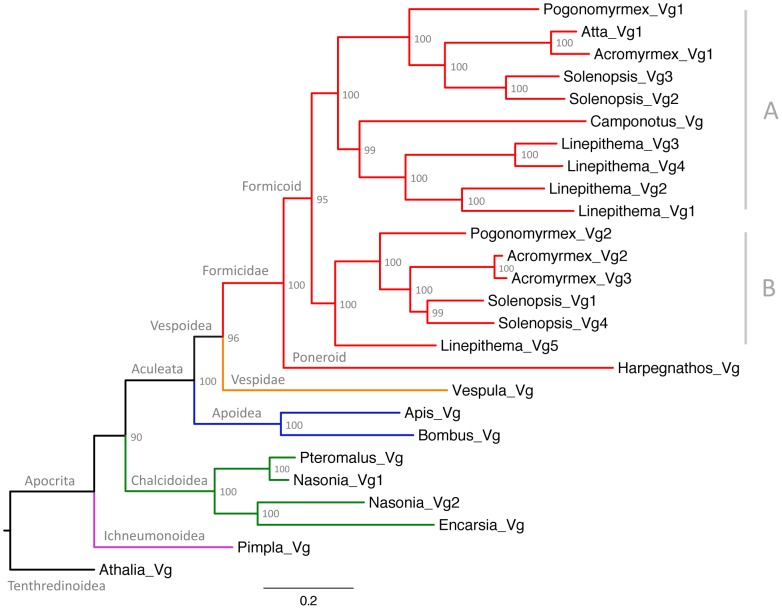
Maximum likelihood tree of ant vitellogenins. Vg sequences from species belonging to representative Hymenopteran groups are also included. Values at the nodes represent bootstrap support from 10,000 replicates. A and B represent different subfamilies of the formicoid clade.

The phylogenetic analysis (Figure 2) revealed that the first duplication of the ancestral Vg gene in ants resulted in two gene subfamilies with different predicted amino acid length (Table 1). The phylogenetic analysis also showed that additional duplications occurred in the lineages leading to *Acromyrmex echinatior*, *Solenopsis invicta* and *Linepithema humile*. Interestingly, the two *Pogonomyrmex barbatus* genes (*Pb_Vg1* and *Pb_Vg2*) respectively cluster with the *S. invicta* genes preferentially expressed in queens (*Si_Vg2* and *Si_Vg3*) and foraging workers (*Si_Vg1* and *Si_Vg4*) [29].

**Figure 3:**
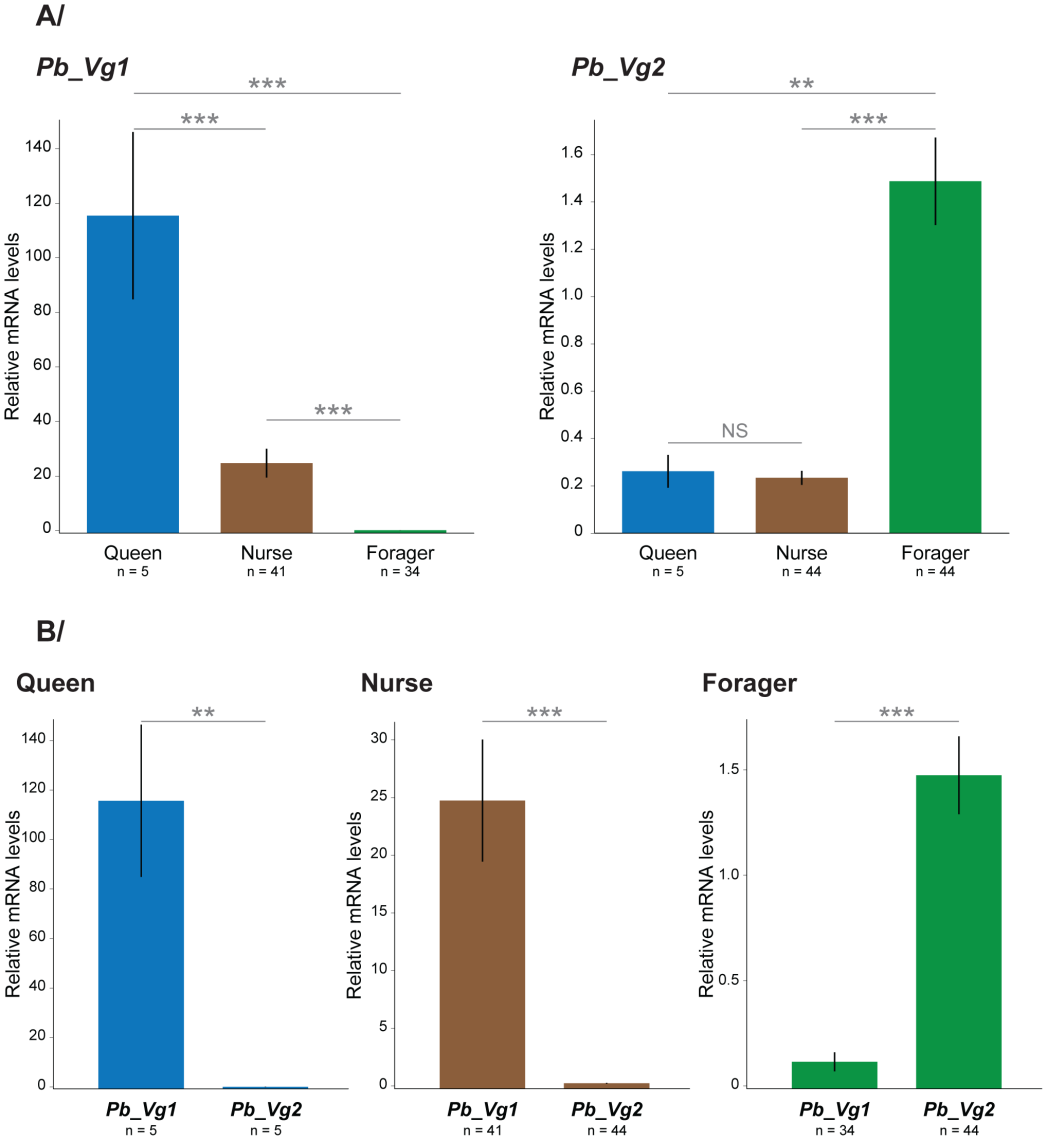
Relative mRNA levels of *Pb_Vg1* and *Pb_Vg2* in queens, nurses and foragers. Values were pooled from five independent colonies (Figure S1). The y axes indicate the relative gene expression, corresponding to the *Pb_Vg1* and *Pb_Vg2* mRNA levels relative to the ribosomal protein RP49 (control) gene mRNA level (mean ± se). The upper panel (A) compares the expression levels of each Vg gene among queens, nurses and foragers. The lower panel (B) compares the expression of both Vg genes separately in queens, nurses and foragers.

To test the prediction that *P. barbatus* Vg genes display caste-specific expression profiles similar to their closest *S. invicta* orthologs, we performed quantitative RT-PCR analysis of Vg genes in *P. barbatus* queens and workers in five independent colonies (Figure S1). On average, *Pb_Vg1* was 4.7 times more highly expressed in queens than in nurses (pMCMC <0.0001) and 908 times more highly expressed in queens than in foragers (pMCMC <0.0001). The expression of *Pb_Vg2* did not differ between queens and nurses (pMCMC = 0.98), but it was on average 5.7 times more highly expressed in foragers than in queens (pMCMC = 0.0028) (Figure 3).

Furthermore, we tested whether the expression of Vg genes in *P. barbatus* is associated to task performance as predicted by the RGPH. We found that *Pb_Vg1* was significantly more highly expressed in nurses than in foragers in 5 out of 5 colonies (Wilcoxon tests; col1: W = 56, p = 0.001; col2: W = 70, p = 0.0001; col3: W = 56, p = 0.0003; col4: W = 49, p = 0.0006; col5: W = 48, p = 0.0007), while *Pb_Vg2* was significantly more highly expressed in foragers than in nurses in 4 out of 5 colonies (Wilcoxon tests; col1: W = 28, p = 0.06; col2: W = 16, p = 0.005; col3: W = 0, p = 0.0003; col4: W = 5, p = 0.02; col5: W = 0, p = 0.0007). On average, *Pb_Vg1* was 190 times more highly expressed in nurses than in foragers (pMCMC<0.0001) and *Pb_Vg2* 6.5 times more highly expressed in foragers than in nurses (pMCMC <0.0001) (Figure 3). *Pb_Vg1* is the predominant transcript in workers as it is expressed in strikingly high levels in nurses compared to *Pb_Vg2* in foragers (Figure 3); a pattern similar to that observed for the single Vg gene in honeybees.

Finally, we performed molecular evolution analyses to determine the relative contributions of selection for novel biochemical functions (i.e. positive selection), selection for the maintenance of existing biochemical functions (i.e. purifying selection) and neutral evolution in the evolution of ant Vg genes. In particular, we estimated selective pressures on two basal branches of Vg respectively leading to primitive ants and modern (Formicoid) ants, and on the two branches that followed the duplication of Vg in the ancestor of modern ants (Figure 2). Analyses of the branch common to the ancestor of all ants (Formicidae) and the branch common to all modern ants yielded identical results: 60.5% of codon sites evolved under purifying selection (dN/dS = 0.24), 39.5% neutrally (dN/dS = 1), and none had evidence for positive selection. The two branches that followed the duplication of Vg are interesting because one branch includes all Vg genes known to be more highly expressed in queens than in workers (hereafter referred to as subfamily A, which includes *Pb_Vg1*, *Si_Vg2* and *Si_Vg3*), while the other branch includes all Vg genes more highly expressed in foraging workers than in queens (hereafter referred to as subfamily B, which includes *Pb_Vg2*, *Si_Vg1* and *Si_Vg4*) (Figure 2 and Table 1). The branch leading to subfamily B shows overall relaxation of purifying selection and no significantly positively selected sites. However, the branch leading to subfamily A shows strong evidence for positive selection (p = 0.008), with a total of 7.1% of sites under positive selection. The three sites with the highest posterior probabilities of being under positive selection in this branch (S44, E382 and N456; pBEB>95%) are part of the main vitellogenin N-terminal lipoprotein domain (LPD-N) that is likely implicated in the uptake of vitellogenin to the ovary [41], providing further support that these changes affect the biochemical properties of the protein.

## Discussion

This study reveals that the genome of *P. barbatus* harbors two Vg paralogs and that Vg underwent one or several rounds of duplication in other species, demonstrating that the existence of multiple Vg genes is a common phenomenon in ants. The phylogenetic analysis clarifies how Vg genes evolve in ants. First, it shows that the first duplication of the ancestral Vg gene occurred after the divergence between the poneroid and formicoid clades. The poneroid clade includes primitive ants of the subfamily Ponerinae while the formicoid clade includes the three main subfamilies of modern ants: Myrmicinae, Dolichoderinae and Formicinae [28], [42]. Because the divergence between primitive and modern ants apparently coincided with the duplication of Vg genes, it is tempting to speculate that this molecular event could have contributed to the evolution of modern ants. Second, the initial pair of Vg paralogs did not experience further duplication or losses in the lineages leading to *Pogonomyrmex barbatus* and *Atta cephalotes* but the ancestor of *Pb_Vg2* appears to be lost in *Camponotus floridanus*. Third, several rounds of duplications occurred independently in the lineages leading to *Solenopsis invicta*, *Linepithema humile* and *Acromyrmex echinatior*, giving rise to multiple Vg genes in each of these species. Intriguingly, the *S. invicta* Vg genes more highly expressed in queens than in workers (*Si_Vg2* and *Si_Vg3*) cluster with *Pb_Vg1* on one branch of the tree, while the genes preferentially expressed in foraging workers (*Si_Vg1* and *Si_Vg4*) cluster with *Pb_Vg2* on the other branch of the tree, suggesting that Vg genes in *P. barbatus* might share a caste-specific expression pattern similar to their closest *S. invicta* orthologs.

This prediction was confirmed by our analyses showing that *Pb_Vg1* is preferentially expressed in queens and *Pb_Vg2* in forager. This suggests that Vg gene duplication and subfunctionalization to acquire caste-specific expression related to reproductive and non-reproductive functions may be a general feature of ant species with multiple Vg genes. Expression and functional analyses in additional species will need to be performed to determine the extent to which this is the case.

Three lines of evidence suggest that the first round of duplication of Vg genes facilitated the evolution of queen-worker specialization. First, the duplication occurred in the common ancestor of formicoids (modern ants). A key feature of these ants is a marked morphological, physiological and behavioral differentiation between queens and workers. This contrasts with non-modern ants that exhibit few or no differences between castes. Second, the gene expression differences we identified suggest that the two subfamilies of Vg paralogs evolved different functions, with genes from subfamily A predominantly playing roles in queens and subfamily B predominantly playing roles in workers. Finally, our molecular evolution analyses suggest that these two subfamilies are evolving differently since the duplication. Positive selection on the paralogs in subfamily A may stem from a process of neofunctionalization associated with the evolution of a dramatically higher reproductive output of queens in modern ant species. Furthermore, relaxation of purifying selection on the subfamily B is consistent with the loss of the reproductive constraints and evolution of new functions of Vg (i.e. subfunctionalization) in workers. These results are thus consistent with the proposal that gene duplication followed by caste-specific expression can circumvent the constraints of antagonistic pleiotropy, thus facilitating the evolution of new caste-specific functions in ants [43], [44]. Subfunctionalization of duplicated genes has been described in other contexts. For example, the duplication of a single copy gene in a basal vertebrate gave rise to oxytocin and vasopressin neurotransmitter genes. These two genes have distinct physiological and behavioral roles in vertebrates while the single homologous gene in invertebrates has both vasopressin-like and oxytocin-like functions [45]. Similarly, duplication of estrogen receptor ER-*β* occurred in the lineage leading to teleost fish. These two copies are expressed in alternate parts of the brain suggesting that subfunctionalization occurred and affects behavior [46].

Interestingly, there is apparently a single copy of Vg in *Apis mellifera* and other bees. A comparative analysis of the evolution of Vg and seven other genes in *A. mellifera* and several closely related species showed evidence of positive selection for Vg [47]. In particular replacement polymorphisms were significantly enriched in parts of the protein involved in binding lipid, suggesting a link between the structure of the gene, its function, and its effects on fitness [47]. These data have been interpreted as social pleiotropy leading to only limited constraints on adaptive protein evolution [47], [48]. This raises the question of why multiple duplications occurred in ants but not in bees. A possible reason lies in the much greater phenotypic differences between queens and workers in ants compared to bees. In many modern ants, queens and workers greatly differ in size and other morphological, physiological and behavioral traits [1], [2]. For example ant workers have lost the ability to fly and in some species they are completely sterile. This higher differentiation in ants than bees may lead to greater selection for different roles of Vg in queens and workers, and thus greater potential benefits for Vg duplication and subfunctionalization in ants. Interestingly, the association between the strength of female caste differentiation and the presence of multiple Vg genes seems to hold in social wasps and termites. Although no genome has been sequenced so far in these two groups of social insects, the available data suggest that the social wasps *Vespula vulgaris* and *Polistes metricus* (low differentiation between castes) only have a single Vg gene [49], [50] while the termite *Reticulitermes flavipes* (high differentiation between castes) have two Vg genes [51].

The pattern of expression of *Pb_Vg1* (high in queens, medium in nurses and low in foragers) is similar to that observed for Vg in the honeybee [25], [52], suggesting an association between ovarian activity and the expression of this gene. In contrast to honeybee nurses which can produce fertile eggs in queenless conditions and sometimes even in the queen presence, workers are sterile in most *Pogonomyrmex* species [32]. However, *Pogonomyrmex* workers can produce trophic eggs which are thought to be the main method of nutrient redistribution because trophallaxis – mouth-to-mouth food transfer - has not been observed in this genus [32]. Interestingly, a recent study in *Pogonomyrmex californicus* showed that nurses had significantly increased ovarian activity compared to foragers [53], suggesting that trophic eggs are specifically produced by nurses. This pattern of nutrient sharing differs from the honeybee, where the proteins and lipids provided to the larvae, queen and foragers come from the hypopharyngeal and mandibular gland secretions of the nurses [53]–[56]. These results, together with our finding that *Pb_Vg1* is predominantly expressed in nurses, suggest that the primary role of this protein as a source of amino acids and lipids for the developing embryo has been co-opted to a novel nutritionally related role associated with the production of trophic eggs. In honeybees Vg expression and ovarian activity are linked with the genetic pathways associated with the regulation of behavior [15], [16]. It remains to be tested if such relation is conserved in sterile ants with functional ovaries that produce trophic eggs.

Our results revealed that the level of expression of *Pb_Vg2* was much lower than that of *Pb_Vg1* in queens. By contrast *Pb_Vg2* was expressed at higher level than *Pb_Vg1* in sterile foragers suggesting that the function of this gene is not related to ovarian activity but likely has a different function in foragers. Our molecular evolution analysis showing overall relaxation of purifying selection and no positively selected sites on the paralogs in subfamily B suggests a new role of these proteins neither associated to its existing biochemical function nor novel functions derived of positively selected domains. Instead, the release of functional constrains on the LPD-N structural domain, implicated in lipid transport and Vg receptor binding [41], suggests that these proteins are not imported to the ovary and after having lost their lipid-binding capabilities, they may be primarily used as source of amino acids in foragers. In honeybees, earlier findings that Vg protein was present not only in fertile queens but also in functionally sterile workers [19], [57] and drones [58] led to the proposal that this yolk protein could have both reproductive and non-reproductive functions [59]. Our results indicate that similar functional division of Vg took place in *P. barbatus* and other modern ant species via gene duplication and subfunctionalization.

The preferential expression of *Pb_Vg1* in nurses and of *Pb_Vg2* in foragers follow a general gene expression pattern observed in honeybees, in which egg-laying workers show up-regulation of genes associated with reproduction while non-reproductive workers overexpress genes involved in foraging-related functions [60]. This is consistent with the RGPH prediction that reproductive pathways were co-opted to regulate worker behavior and supports the evolutionary theory prediction that potentially reproductive individuals are selected to carry out low-risk tasks, in order to not compromise their reproductive future [61], [62]. In ants, this ancestral mechanism regulating division of labor has been conserved, even in species with workers having lost their reproductive potential.

### Conclusion

The results of this study suggest that Vg has been co-opted to regulate worker behavior in the ant *P. barbatus* as in the honeybee. Support for RGPH in groups of insects that evolved sociality independently, demonstrates that the co-option of reproductive pathways to regulate behavior is a major director in the evolution of sociality in insects. On the other hand, the expression of one Vg paralogs in sterile workers reveals that Vg adaptation to regulate worker behavior is not necessarily linked to reproduction but maybe linked exclusively to nutritional functions. Our result suggests that, after the initial duplication in ants, *Vg* genes underwent neo- or subfunctionalization to acquire caste- and behavioral-specific functions. Overall, our results suggest that even though ants and bees evolved sociality independently, they have conserved similar mechanisms to regulate division of labor.

## Materials and Methods

### Gene prediction and annotation

To determine gene models, we first ran TBLASTN using known hymenopteran Vg sequences against ant genome sequences downloaded from the fourmidable database [63]. Subsequently, ruby/bioruby scripts [64] were used to extract relevant subsets of each genome. Gene predictions were generated on each subset using MAKER2 [65] s65mand subsequently manually refined using Apollo [66]. Conflicts gene predictions were resolved by using EST data when available, splice prediction algorithms (http://www.fruitfly.org/seq_tools/splice.html) and manual verification of splicing consensus sequences.)

### Phylogenetic analyses

Inaccurate sequence alignment or phylogeny leads to misleading or incorrect results in molecular evolution analyses. We used an approach centered on the use of phylogenetically aware codon-level aligner PRANK, which is likely to minimize the risks of introducing errors [67], [68]. This required several steps. We preliminarily aligned the 26 Vg protein sequences with MAFFT linsi [69] and removed ambiguous sections of the alignment using trimAl (option -gappyout) [70]. A first tree was built with RAxML (model GTRGAMMAI) [71] and rooted with “nw_root” (Newick Utilities package [72]). This tree was used as a guide tree in PRANK [73] to obtain a high-quality codon-level alignment of the 26 Vg coding sequences. Ambiguous sections of the alignment were removed using trimAl (option -gappyout) and a final tree was built with RAxML (GTRGAMMAI model); 10,000 bootstraps were generated to assess its confidence. Selective pressures (dN/dS) on different parts of the phylogenetic tree were estimated using the branch-site codon-substitution model from CodeML (PAML 4.6) [74]. Such dN/dS ratios are obtained by computing the number of non-synonymous changes (dN) over synonymous changes (dS) (see Table 2 for more details). Vg site coordinates (S44, E382, N456) are given as in *Apis mellifera* Vg (Uniprot identifier VIT_APIME).

**Table 2:** Results of test of positive selection.

Branch	lnL0	lnL1	2*(lnL1-lnL0) [p-value, df = 1]	ω0	p0 (%)	ω1	p1 (%)	ω2	p2a + p2b(%)	Sites BEB>95%
Formicidae	−62600.98	−62600.98	0.00 [1.000]	0.24	60.5	1.00	39.5	1.00	0	None
Formicoid	−62600.98	−62600.98	0.00 [1.000]	0.24	60.5	1.00	39.5	1.00	0	None
Subfamily A	−62597.90	−62594.35	7.12 [0.008**]	0.24	56.5	1.00	36.4	15.15	7.1	S44 (87), E382 (761), N456 (963)
Subfamily B	−62600.29	−62598.65	3.29 [0.070]	0.24	59.5	1.00	38.9	17.80	1.5	None

Selective pressures are divided into three classes: ω0 (dN/dS<1, negative selection), ω1 (dN/dS = 1, neutral evolution) and ω2 (dN/dS ≥1, positive selection). p0, p1 and p2a+p2b are their respective proportion. lnL0 and lnL1 are the likelihood values of the null (ω2 = 1) and alternative (ω2≥1) models, respectively. The likelihood ratio test [2*(lnL1-lnL0)] allows discriminating between neutral evolution and positive selection. A p-value<0.01 presents a significant test (marked as **). Sites under positive selection with BEB score >95% are shown.

### Sample collection

*Pogonomyrmex barbatus* founding queens were collected during nuptial flights on July 15^th^, 2008 in Bowie, Arizona, USA (N32°18′54″//W109°29′03″). Colonies were then kept in laboratory conditions (30°C, 60% humidity and 12 h/12 h light∶dark cycle) in 15×13×5 cm plastic boxes with water tubes, and were fed once a week with grass seeds and a mixture of eggs, honey and smashed mealworms. Thirty months later, five of these colonies were used to collect samples on December 16^th^, 2010. Task performance in workers is age related, thus nurses tend to be younger than foragers [2]. Young ants interacting with the brood in the nest tube were considered as nurses. To collect foragers, each colony was connected with a cardboard-made bridge to a foraging area composed of a plastic box containing grass seeds. Any ant handling a food item in the foraging area was considered as forager. Ant samples were flash-frozen in liquid nitrogen and kept at −80°C for further RNA extraction.

### Gene expression analysis

Whole body worker samples were used to measure the expression of *Pb_Vg1* and *Pb_Vg2* genes. RNA extractions were performed using a modified protocol including the use of Trizol (Invitrogen) for the initial homogenization step, RNeasy extraction kit and DNAse I (Qiagen) treatment to remove genomic DNA traces. For each individual worker, cDNAs were synthesized using 500 ng of total RNA, random hexamers and Applied Biosystems reagents. Levels of mRNA were quantified by quantitative real-time polymerase chain reaction (qRT-PCR) using ABI Prism 7900 sequence detector and SYBR green. All qPCR assays were performed in triplicates and subject to the heat-dissociation protocol following the final cycle of the qPCR in order to check for amplification specificity. qRT-PCR values of each gene were normalized by using an internal control gene (RP49). Paralog-specific primers (Table S1) were designed using sequence alignment [75] and primer analysis [76] programs. Primer sequences overlapped coding regions split by introns, allowing the specific amplification of cDNA levels over eventual genomic DNA contaminations. Transcript quantification calculations were performed by using the ΔΔCT method [77].

### Statistical analysis

All data were analyzed using R (http://www.r-project.org/) and the R packages lme4 [78] and language R [79]. The effect of caste on gene expression relative values was analyzed using linear mixed effects models. To avoid pseudoreplication, the colony was included as a random effect. We checked for normality and homogeneity by visual inspections of plots of residuals against fitted values. To assess the validity of the mixed effects analyses, we performed likelihood ratio tests to confirm that the models with fixed effects differed significantly from the null models with only the random effects. Throughout the paper, we present MCMC (Markov-chain Monte Carlo) estimated p-values that are considered significant below the 0.05 threshold (all significant results remained significant after Bonferroni correction).

## Supporting Information

Figure S1 Relative mRNA levels of *Pb_Vg1* and *Pb_Vg2* in queens, nurses, and foragers. Experiments were performed in five independent colonies of *Pogonomyrmex barbatus*. The y axes indicate the relative gene expression, corresponding to the *Pb_Vg1* (panel A) and *Pb_Vg2* (panel B) mRNA levels relative to the ribosomal protein RP49 (control) gene mRNA level (mean ± se).
(TIF)

Table S1 Paralog-specific primers used for qRT-PCR (5′-3′ order).
(DOCX)
