# Supplementary figures and images for: Vitellogenin Underwent Subfunctionalization to Acquire Caste and Behavioral Specific Expression in the Harvester Ant Pogonomyrmex barbatus
Source: PLoS Genet. 2013 Aug 15;9(8):e1003730. doi: 10.1371/journal.pgen.1003730 (PMC3744404; doi:10.1371/journal.pgen.1003730)

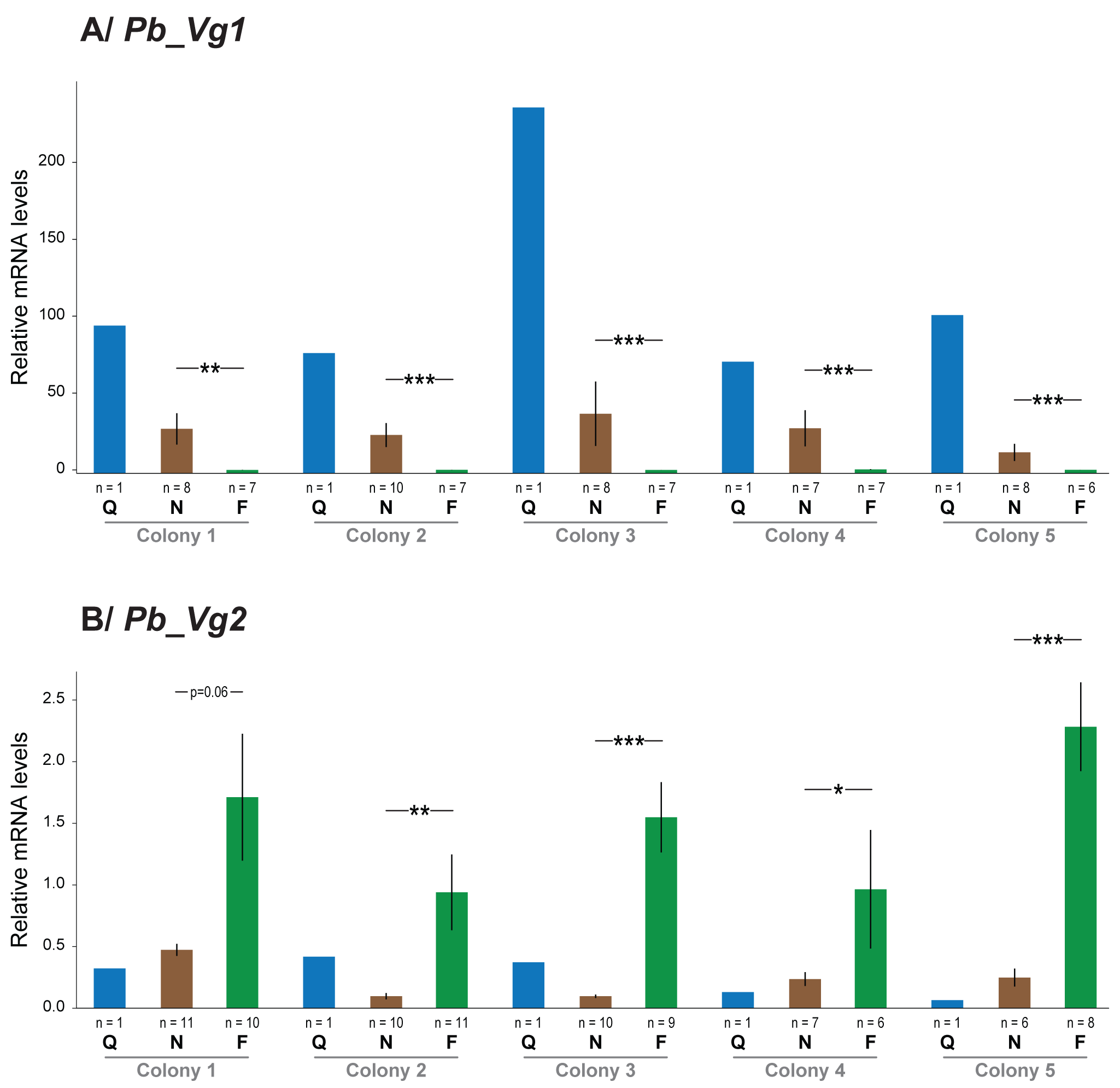

Supplement: Figure S1 — Relative mRNA levels of Pb_Vg1 and Pb_Vg2 in queens, nurses, and foragers. Experiments were performed in five independent colonies of Pogonomyrmex barbatus. The y axes indicate the relative gene expression, corresponding to the Pb_Vg1 (panel A) and Pb_Vg2 (panel B) mRNA levels relative to the ribosomal protein RP49 (control) gene mRNA level (mean ± se). (TIF) [file pgen.1003730.s001.tif]
