# Supplementary material for: Vitellogenin Underwent Subfunctionalization to Acquire Caste and Behavioral Specific Expression in the Harvester Ant Pogonomyrmex barbatus
Source: PLoS Genet. 2013 Aug 15;9(8):e1003730. doi: 10.1371/journal.pgen.1003730 (PMC3744404; doi:10.1371/journal.pgen.1003730)
Supplement: Table S1 — Paralog-specific primers used for qRT-PCR (5′-3′ order). (DOCX) [file pgen.1003730.s002.docx]

|  | Forward | Reverse |
| --- | --- | --- |
| Vg1 | ACAGGACGATGTTGTTTCGGAATTA | TCGTCACGGATGATTGAATGGTATAT |
| Vg2 | TCTAATGATGGAGTTCTTTCGAGATCA | ACGGAAGACTGAATAGTGAAGCGTT |
| RP49 | CGATAGATATGACAAACTCAAACGCAAC | GTATTGGCCCTTGAAACGTCTGCG |
